# Glucose metabolism impairment as a hallmark of progressive myoclonus epilepsies: a focus on neuronal ceroid lipofuscinoses

**DOI:** 10.3389/fncel.2024.1445003

**Published:** 2024-09-19

**Authors:** Lorenzo Santucci, Sara Bernardi, Rachele Vivarelli, Filippo Maria Santorelli, Maria Marchese

**Affiliations:** ^1^Neurobiology and Molecular Medicine Unit, IRCCS Fondazione Stella Maris, Calambrone, Italy; ^2^Department of Biology, University of Pisa, Pisa, Italy

**Keywords:** glucose metabolism, neurodegeneration, progressive myoclonic epilepsies, neuronal ceroid lipofuscinosis, anti-diabetics

## Abstract

Glucose is the brain’s main fuel source, used in both energy and molecular production. Impaired glucose metabolism is associated with adult and pediatric neurodegenerative diseases such as Alzheimer’s disease (AD), Parkinson’s disease (PD), GLUT1 deficiency syndrome, and progressive myoclonus epilepsies (PMEs). PMEs, a group of neurological disorders typical of childhood and adolescence, account for 1% of all epileptic diseases in this population worldwide. Diffuse glucose hypometabolism is observed in the brains of patients affected by PMEs such as Lafora disease (LD), dentatorubral-pallidoluysian (DRPLA) atrophy, Unverricht–Lundborg disease (ULD), and myoclonus epilepsy with ragged red fibers (MERRFs). PMEs also include neuronal ceroid lipofuscinoses (NCLs), a subgroup in which lysosomal and autophagy dysfunction leads to progressive loss of vision, brain atrophy, and cognitive decline. We examine the role of impaired glucose metabolism in neurodegenerative diseases, particularly in the NCLs. Our literature review, which includes findings from case reports and animal studies, reveals that glucose hypometabolism is still poorly characterized both *in vitro* and *in vivo* in the different NCLs. Better identification of the glucose metabolism pathway impaired in the NCLs may open new avenues for evaluating the therapeutic potential of anti-diabetic agents in this population and thus raise the prospect of a therapeutic approach able to delay or even halt disease progression.

## 1 Introduction

Glucose is the brain’s main fuel source (Jais et al., 2016), primarily used to produce energy in the form of adenosine triphosphate (ATP), which is needed for maintaining normal neuronal cell function. Approximately 70% of the ATP produced is used for signaling activities such as action potential generation and synaptic transmission, while the remaining 30% is used for non-signaling activities such as axonal transport, biosynthesis, and other basic cell functions (Zhang et al., 2021). Studies evaluating the effects of low levels of glycemia confirm that glucose is the brain’s primary energy source. In insulin-induced hypoglycemia, symptoms ranging from mild sensory disturbance to lethargy and even coma were reversed by administering glucose, while the administration of compounds such as glycerol, lactate, and pyruvate did not improve the symptoms (Clarke and Sokoloff, 1999). However, the brain can also use ketone bodies (acetoacetate and β-hydroxybutyrate) as an energy source in certain critical conditions, such as fasting, physical stress, and starvation (Bowman et al., 2019). Glucose is stored as glycogen in liver, muscle, and brain tissue, principally in astrocytes and, to a lesser extent, in neurons (Carlson et al., 2018). It is not surprising, therefore, that neurodegeneration is associated with impaired glucose metabolism. Neurodegenerative diseases such as AD and PD are associated with glucose hypometabolism in the brain (Tang, 2020). In AD, positron emission tomography (PET) with [^18^F]2-fluoro-2-deoxy-D-glucose ([^18^F]DOG) showed reduced glucose metabolism in the regions affected by the pathological changes associated with the disease, such as the hippocampus and frontal, temporal, parietal, occipital, and entorhinal cortices (Heiss et al., 1991). AD patients also showed reduced expression of GLUT1 and GLUT3 in the hippocampus and cerebral cortex (Simpson et al., 1994). This finding may be associated with the downregulation of β-catenin and modifications of hypoxia-inducible factor 1α, which also affect the expression of the PI3K/Akt pathway in AD (Yue et al., 2010).

Another neurological disorder linked to decreased GLUT1 expression or function is GLUT1 deficiency syndrome (Benarroch, 2014). This condition, associated with pathogenic variants in *SLC2A1* (Hewson et al., 2018), is characterized by symptoms such as intellectual impairment, microcephaly, epilepsy, and movement disorders (Brockmann, 2009). These symptoms have been found to drastically improve once patients are started on a ketogenic diet (Hewson et al., 2018).

Reduced glucose metabolism is also observed in PMEs, a group of diseases that includes DRPLA (Sone et al., 2016), LD (Jennesson et al., 2010), ULD (Muccioli et al., 2022), MERRFs (Berkovic et al., 1989), and the NCLs (Jennesson et al., 2010; De Volder et al., 1990; Kitani et al., 1995), the latter characterized by epilepsy, retinal changes, dementia, and brain atrophy (Zimmern and Minassian, 2024).

This review looks at the impact of impaired glucose metabolism on brain function in the PMEs, especially the NCLs, and shows it to be a hallmark of these conditions.

## 2 Materials and methods

A PubMed database search was conducted using two queries: <<glucose [All Fields]> > AND < <progressive myoclonus epileps*[All Fields]> > and < <glucose [All Fields]> > AND < <neuronal ceroid lipofuscinosi*[All Fields]>>. Selected articles had to be full-text articles, written in English and published by 5 March 2024. The two queries identified 20 and 17 articles, respectively. These articles were then manually filtered to select only those containing information relevant to this review. Additional articles were identified from the reference lists of the selected articles or through other methods, such as website searches and manual searches.

**Figure 1:**
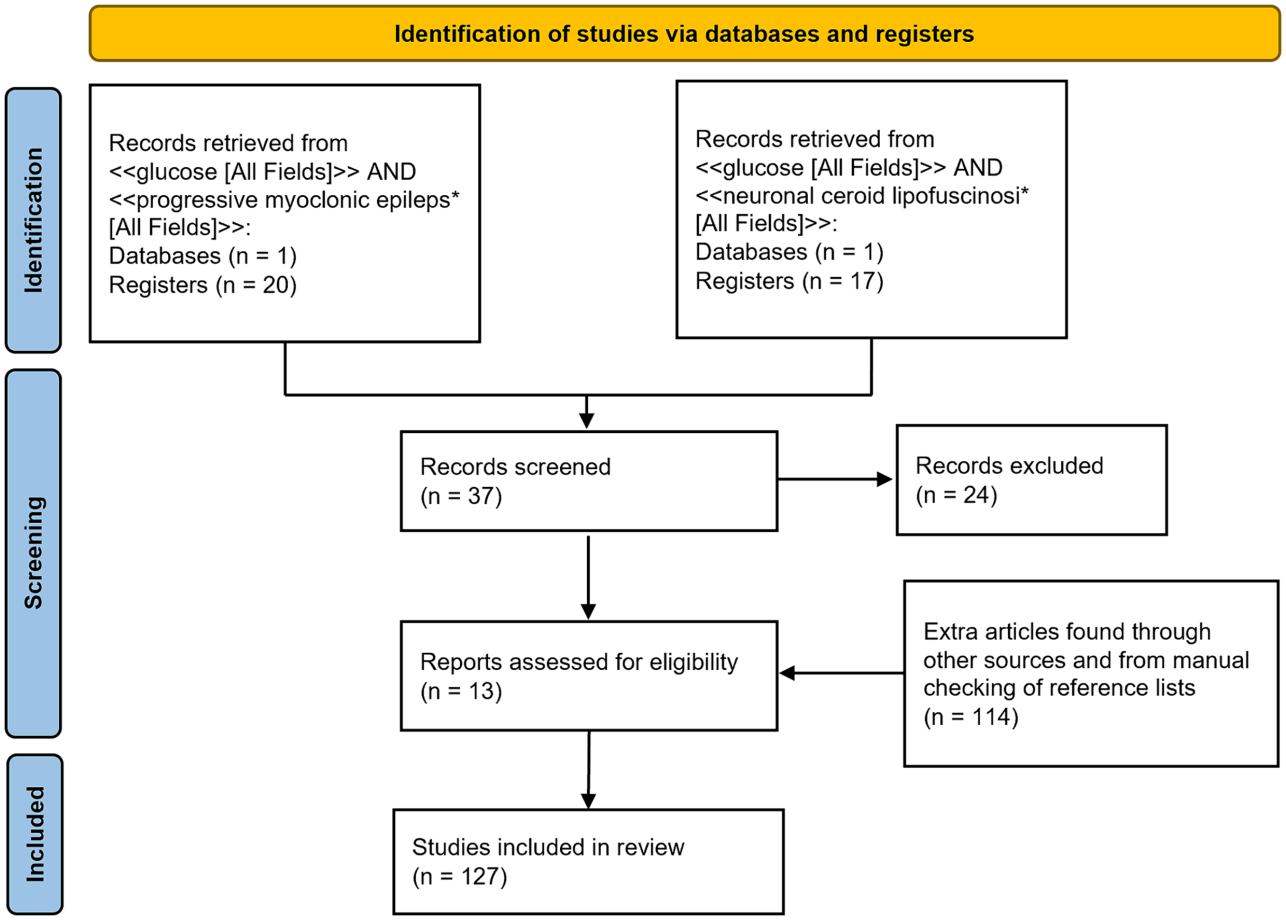
PRISMA 2020 flow diagram of the literature search progress.

Figure 1 shows a PRISMA flow diagram (Page et al., 2021) summarizing the methodology.

## 3 Glucose in the brain

### 3.1 How glucose is transported in the brain

Glucose enters neuronal cells through a process of facilitated diffusion involving different types of transporters. Most of these transporters belong to the solute carrier 2 family (GLUT transporters) or the solute carrier 5 family (Na^+^-D-glucose co-transporters). Since glucose is the brain’s primary energy source, its transport and uptake must be precisely regulated (Koepsell, 2020). Among the main glucose transporters, we considered the following (also listed in Table 1):

*GLUT1*: In the blood–brain barrier, glucose leaves blood vessels through the GLUT1 present in the luminal and abluminal membranes of endothelial cells. GLUT1 is a high-affinity transporter that exploits the difference in glucose concentration between the blood and the brain. Normally, the glucose concentration in the blood is approximately 4 to 6 mM (Patching, 2016), while in the brain interstitium, it is 1–2 mM (Gruetter et al., 1998). This difference, along with the metabolic degradation of glucose by neuronal cells, creates a force that draws glucose out of the blood circulation. GLUT1 is also expressed in other cell types, such as astrocytes. These cells come into contact with the blood vessels via the permeable gap junctions in their endfeet, where the transporter is located (Alvarez et al., 2013). Once inside astrocytes, glucose can be converted into L-lactate or transferred to neurons via GLUT1. This transporter can also carry D-glucose, D-galactose, D-glucosamine, and glucose analogs such as 2-deoxy-D-glucose (2DOG) (Koepsell, 2020).
*GLUT2*: GLUT2 is a low-affinity transporter of D-glucose, D-galactose, D-fructose, and 2DOG. It also functions as a glucose receptor capable of upregulating its expression (Guillemain et al., 2000). As well as in neurons, astrocytes, and oligodendrocytes, GLUT2 can be found in pancreatic β cells and hepatocytes (Arluison et al., 2004). It is believed to be involved in the regulation of food and glucose intake and the regulation of glucose homeostasis in the central nervous system (Wan et al., 1998).
*GLUT3*: GLUT3 is a widely expressed glucose transporter in the brain (Mantych et al., 1992). It is more efficient than GLUT1 but only transports D-glucose. GLUT3 is highly expressed in all the different cellular compartments of neurons (Gerhart et al., 1992), and it is thought to be used for the uptake of glucose for normal neuronal function and survival.
*GLUT4*: GLUT4 is an insulin-sensitive glucose transporter often co-expressed with GLUT3 in neurons (Apelt et al., 1999). Increased energy demand associated with conditions of high neuronal activity (active neuronal circuits or firing neurons) is met by the insertion of GLUT4 in the axonal plasma membrane under the control of AMP-activated kinase (Ashrafi et al., 2017).
*GLUT5*: GLUT5 is a selective transporter of D-fructose expressed in brain microglial and microvascular endothelial cells (Maher et al., 1994; Mantych et al., 1993).
*GLUT6*: GLUT6 is a low-affinity D-glucose transporter (Joost and Thorens, 2001). It is located in intracellular compartments such as lysosomes, where it does not transport glucose but seems to be involved in modulating inflammatory responses (Maedera et al., 2019). The physiological and pathological functions of GLUT6 remain poorly understood.
*GLUT8*: GLUT8 is ubiquitously expressed in the human body, including the brain (Doege et al., 2000). Its pathophysiological functions are not fully understood. However, a knockout mouse model showed a hyperactive phenotype without effects on memory or explorative behavior, suggesting that this transporter may be associated with energy supply for hippocampal neurons (Schmidt et al., 2008).
*SLC5A1*: SLC5A1 is a secondary active co-transporter of D-glucose that co-transports two Na^+^ ions and one glucose molecule. It is expressed in glial cells, neurons, and capillary endothelial cells and is thought to draw glucose from the interstitium into these cell types, serving as a mechanism against possible glucotoxicity (Koepsell, 2020). The pathological role of SLC5A1 is not well known, but cognitive impairment and damage to hippocampal neurons after chronic brain hypoperfusion were reduced in *Slc5a1* knockout mice (Ishida et al., 2020). SLC5A1 reduction also seems to have a protective role in traumatic brain injury (Sebastiani et al., 2018).

**Table 1:** Distribution and molecular specificity of GLUT glucose transporters in neuronal cells.

Isoform	Expression in brain cells	Molecular specificity	References
GLUT1	Astrocytes (high), neurons, oligodendrocytes, microglial cells	D-glucose, D-galactose, D-glucosamine, and the glucose analogs such as 2-deoxy-D-glucose (2DOG)	Koepsell (2020), Maher et al. (1994), and Wang et al. (2019)
GLUT2	Neurons, astrocytes, oligodendrocytes	D-glucose, D-galactose, D-fructose, 2DOG	Gould et al. (1991) and Burant and Bell (1992)
GLUT3	Neurons (high), astrocytes, microglial cells	Principally D-glucose, but also D-galactose and 2DOG	Gerhart et al. (1992), Apelt et al. (1999), and Burant and Bell (1992)
GLUT4	Neurons, astrocytes, microglial cells	D-glucose, D-galactose, 2DOG	Apelt et al. (1999), Ashrafi et al. (2017), and Burant and Bell (1992)
GLUT5	Neurons, microglial cells	Selective for D-fructose	Maher et al. (1994) and Mantych et al. (1993)
GLUT6	Localization in specific cellular-type is poorly understood	Low affinity for D-glucose and 2DOG	Joost and Thorens (2001) and Maedera et al. (2019)
GLUT8	Astrocytes, microglial cells	Low affinity for D-glucose and 2DOG, but poorly understood	Doege et al. (2000) and Cornford et al. (2000)
SLC5A1	Neurons, astrocytes	D-glucose, D-galactose, 2DOG	Wright et al. (2011) and Balen et al. (2008)

### 3.2 How glucose is regulated in the brain

When neuronal activity increases—during sensory stimulation, mental activities, or exercise—neurons require more energy to sustain the additional synaptic transmission (Harris et al., 2012). This demand for energy translates into a need for more glucose. The increased influx of glucose is facilitated by changes in the characteristics of the blood–brain barrier, as well as changes in neurons and glial cells (Bertossi et al., 1999). However, even though more glucose is reaching the brain, its concentration in the interstitium remains unchanged because the speed at which it enters the cells and is phosphorylated increases, maintaining the gradient.

During neuronal activation, membrane expression of GLUT1 in astrocytes rapidly increases (Boado et al., 1999). Similarly, GLUT1 in the plasma membrane of brain endothelial cells is upregulated, likely through paracrine activation by astrocytes (Boado et al., 1999). This increased GLUT1 expression leads to a high L-lactate concentration (Caesar et al., 2008). Under conditions of increased energy demand, subpopulations of hippocampal neurons and neuronal progenitor cells produce and release insulin to quickly increase the expression of GLUT4 (Csajbók and Tamás, 2016; Kuwabara et al., 2011), thereby allowing a higher level of glucose uptake by neurons. Additionally, in high-energy-demand situations, the membrane abundance of GLUT3 in neurons can also increase (Ashrafi et al., 2017).

Signal molecules can also regulate glucose metabolism in the brain. For instance, the Wnt pathway plays a significant role in the central nervous system, influencing processes, such as development and the modulation of functions like mitochondrial dynamics (Cisternas et al., 2016). The Wnt ligand Wnt3a can enhance glucose uptake by upregulating hexokinase activity, which is linked to the activation of AMPK, resulting in an increased glycolytic rate (Cisternas et al., 2016). Insulin also modulates brain glucose metabolism by upregulating the expression of GLUT3 and GLUT4 in the membrane via the PI3K/AKT pathway (Apelt et al., 1999; Cisternas et al., 2016).

Glucose can also serve as a source of carbon and hydrogen for producing other biological molecules, such as in the synthesis of amino acids, nucleotides, glycogen, lipidic acids, glycolipids, glycoproteins, neurotransmitters, and neuromodulators (GABA, glutamate, aspartate, acetylcholine, D-serine, and glycine), for example (Camandola and Mattson, 2017; Dienel, 2019).

### 3.3 How glucose acts locoregionally and temporally in the brain

**Figure 2:**
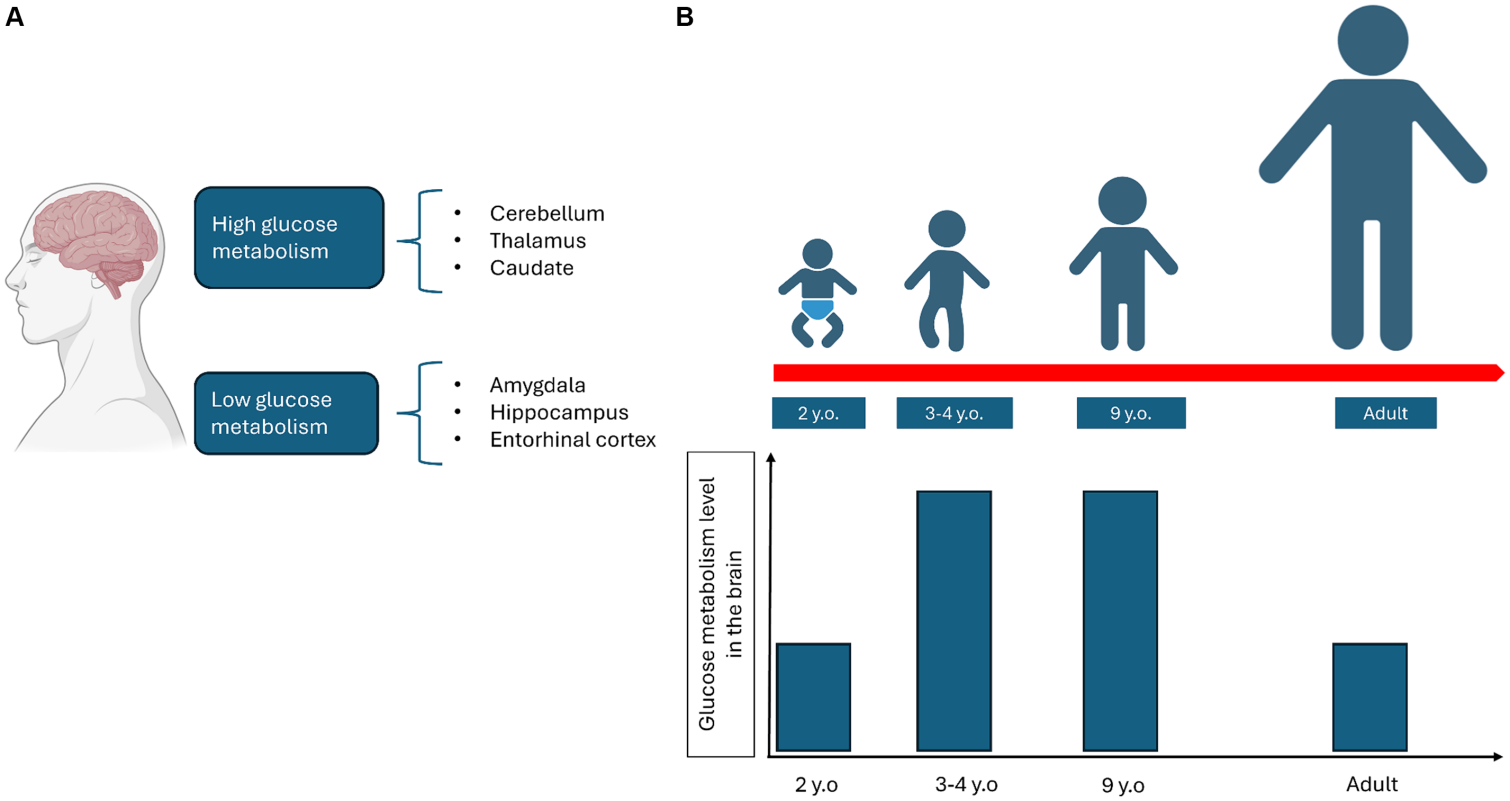
Representation of how glucose metabolism varies depending on the different areas of the brain **(A)** and during the growth process **(B)**.

There is extensive literature on the different utilization of glucose in distinct areas of the brain and during development. PET studies have identified brain regions with varying levels of glucose metabolism (Tomasi et al., 2017). For example, the amygdala, hippocampus, and entorhinal cortex exhibit low glucose metabolism, while other areas, such as the cerebellum, thalamus, and caudate, show high glucose metabolism (Tomasi et al., 2017) (Figure 2A). In the brains of young adults aged 20–33 years, aerobic glycolysis is predominant in the prefrontal, medial parietal, and lateral cortices, but is less present in the cerebellum and medial temporal lobes (Vaishnavi et al., 2010). During brain development, the adult cerebral metabolic rate of glucose values is reached at 2 years of age. They then continue to rise to reach twice the adult levels at 3–4 years of age and remain stable until 9 years of age. After that, they begin to decline, returning to normal adult levels (Chugani et al., 1987). The cerebral metabolic rate of oxygen in 6-year-old children is 25% higher than in young adults (Kennedy and Sokoloff, 1957), while total daily glucose utilization peaks at 4–5 years of age (Kuzawa et al., 2014) (Figure 2B).

### 3.4 Glucose metabolism in neuronal cells

**Figure 3:**
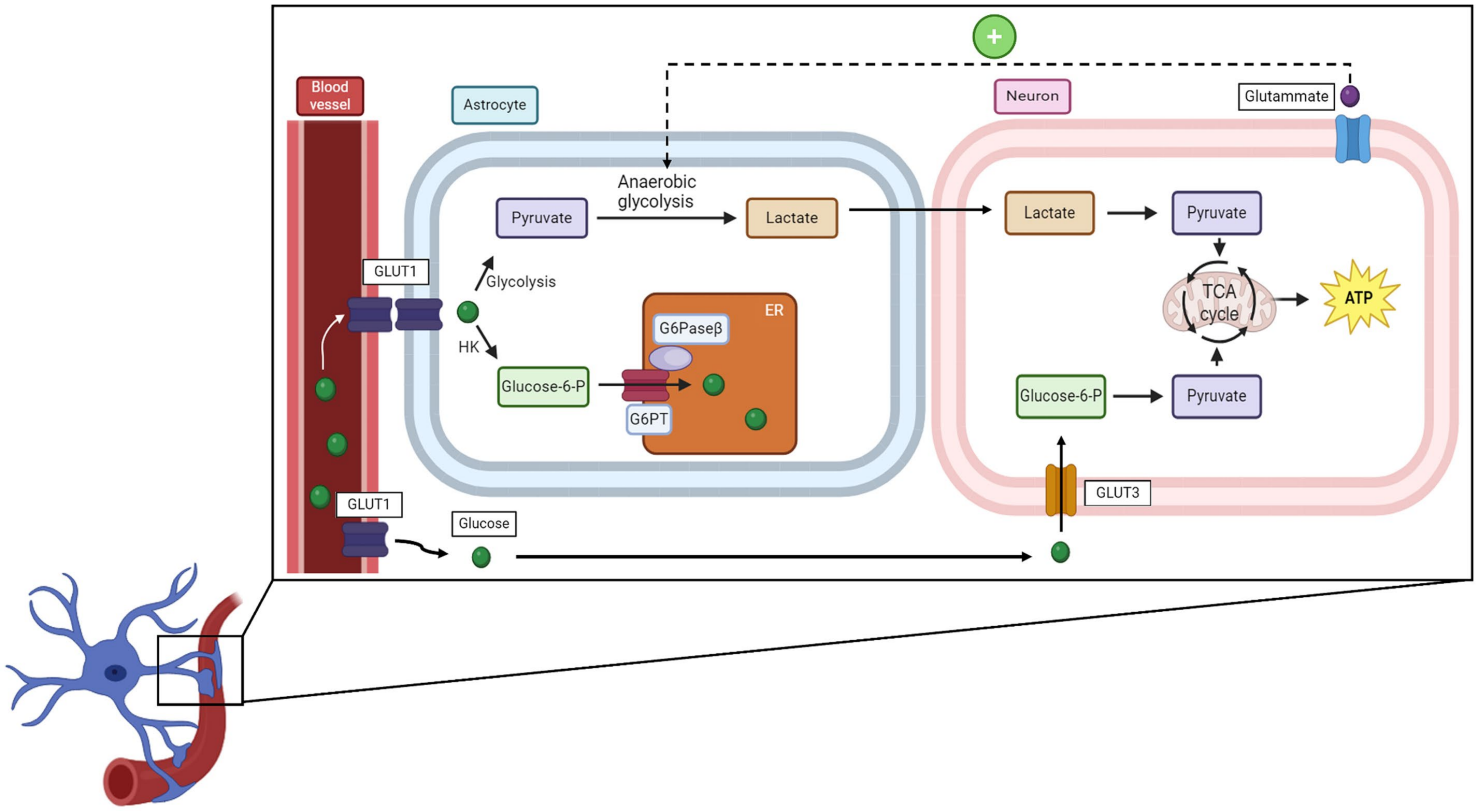
How glucose is uptaken by neurons and the relationship between neurons and microglial cells. HK, hexose kinase; G6PT, glucose-6-phosphate translocase; G6Paseβ, glucose-6-phosphatase-β; TCA cycle, tricarboxylic acid cycle.

In neuronal cells, including excitatory and inhibitory neurons as well as glial cells, glucose is used both for non-signaling activities and for specific processes such as synaptic transmission, action potential, and recycling of neurotransmitters or calcium responses (Yu et al., 2018). Glucose metabolism differs between neurons and astrocytes. Astrocytes, via their endfeet, come into close contact with blood vessels, which facilitates glucose uptake by GLUT1 transporters (Alvarez et al., 2013). Once inside astrocytes, glucose is rapidly phosphorylated to glucose-6-phosphate, which traps it within the cell. Then, it is translocated into the endoplasmic reticulum (ER) through the combined action of glucose-6-phosphatase-β, an enzyme specifically expressed in the astrocyte ER that removes the previously added phosphate and glucose-6-phosphate translocase, which transfers glucose from the cytosol into the ER lumen. This process allows for rapid glucose transfer to neurons (Müller et al., 2018). Neurons, having higher levels of hexokinase, phosphorylate glucose faster than other cells, suggesting that glucose is preferentially taken up by them (Lundgaard et al., 2015). In neurons, glucose is used primarily for oxidative phosphorylation, while in astrocytes, anaerobic glycolysis is the predominant mechanism due to the higher levels of lactate dehydrogenase (Halim et al., 2010). Astrocytes and neurons are associated with the astrocyte-neuron lactate shuttle hypothesis: when astrocytes are stimulated by the glutamate released from highly firing neurons, they convert a greater quantity of glucose into lactate through anaerobic glycolysis. This lactate is then transferred into neurons, where it will be used as an energy source. During high activity, neurons can also switch to lactate production to obtain more energy and therefore do not necessarily depend on the astrocytes (Bélanger et al., 2011). However, a recent study indicates that neurons can also directly uptake glucose for glycolysis and, thus for energy production (Li et al., 2023). A schematic representation is provided in Figure 3.

Astrocytes can store glucose in the form of glycogen granules, with the quantity of these granules reflecting the activity of the specific brain region; higher levels are found in high-energy-demand zones (Oe et al., 2016). It is hypothesized that glycogen in astrocytes serves as a glucose source for use in critical conditions, such as starvation and stress (Dienel, 2019). The fact that impaired glycogen metabolism is associated with neurological diseases such as epilepsy and AD underlines the importance of glucose storage in the form of glycogen (Lk et al., 2018). Energy obtained from glycogen degradation is used to remove glutamate and potassium from the synaptic gap, and if glycogen metabolism is altered, these components cannot be removed, leading to hyperexcitability (Kilic et al., 2018). Conversely, excessive accumulation of glycogen can cause LD (Nitschke et al., 2018).

## 4 Impaired glucose metabolism in progressive myoclonic epilepsies

The PMEs are a group of rare neurodegenerative conditions (Bernardi et al., 2023). Examples include diseases associated with abnormal cellular storage (LD and NCLs), mitochondrial dysfunction (MERRF), or brain degeneration (DRPLA) (Bernardi et al., 2023). The symptoms of PMEs include epilepsy, ataxia, and cognitive impairment (Bernardi et al., 2023). Another common clinical hallmark is reduced glucose metabolism in the brain, as will be discussed in the following paragraphs. PMEs are debilitating, and their symptoms are generally resistant to treatment. They are estimated to account for up to 1% of all epileptic diseases in children and adolescents worldwide (Orsini et al., 2019). In this review, we will focus mainly on NCLs but will first briefly comment on other less common forms of PME.

### 4.1 Lafora disease (LD)

LD is an autosomal recessive form of PME characterized by the presence of so-called Lafora bodies composed of polyglucans (Jennesson et al., 2010) that can be found in patient’s skin biopsies. Symptoms include generalized seizures, visual disturbances, and progressive cognitive impairment (Serratosa et al., 2012). More than 95% of patients present mutations in two genes associated with glycogen metabolism: *EPM2A*, which codes for laforin, and *EPM2B*, which codes malin (Serratosa et al., 2012). PET scans using [^18^F]DOG in LD patients have shown hypometabolism in different regions of the brain, particularly in the occipital lobe (Jennesson et al., 2010).

### 4.2 Dentatorubral-pallidoluysian atrophy (DRPLA)

In DRPLA, an abnormal expansion of CAG repeats in *atrophin-1* gene causes autosomal dominant hereditary spinocerebellar degeneration, characterized by symptoms such as myoclonus, epilepsy, ataxia, and dementia (Sone et al., 2016). Magnetic resonance imaging in DRPLA patients has shown diffused atrophy in the brain, cerebellum, and brainstem (Sone et al., 2016), while PET images with [^18^F]DOG revealed glucose hypometabolism in the striatum, with the extent of the reduction seemingly correlated with the number of CAG repeats (Sone et al., 2016).

### 4.3 Unverricht–Lundborg disease

ULD is a neurodegenerative disorder with onset in late childhood or adolescence. It is caused by cysteine protease inhibitor cystatin B (*CSTB*) mutation, and it is transmitted as an autosomal recessive trait. The disease is characterized by stimulus-sensitive myoclonus, tonic–clonic epileptic seizures, ataxia, intentional tremor, and mild cognitive decline (Kälviäinen et al., 2008). [^18^F]DOG-PET shows hypometabolism, especially in the thalami, frontal and parietal lobes, and posterior brainstem (Muccioli et al., 2022).

### 4.4 Myoclonus epilepsy with ragged red fibers (MERRFs)

MERRFs are a multisystem mitochondrial syndrome associated with a mutation in mitochondrial DNA of the tRNA (Lys) gene (Hameed and Tadi, 2023). The symptoms include myopathy, cerebellar ataxia, cardiac arrhythmia, optic atrophy, and dementia (Hameed and Tadi, 2023). The pathophysiology of MERRF is believed to be associated with alteration of the mitochondrial respiratory chain due to the tRNA mutation, resulting in decreased cellular energy, ion-channel malfunction, and ultimately neuronal death (Hameed and Tadi, 2023). [^18^F]DOG-PET scans in MERRF patients revealed a significant reduction in cortical glucose metabolism, likely due to the impaired mitochondrial respiratory chain (Berkovic et al., 1989).

## 5 Neuronal ceroid lipofuscinoses (NCLs)

NCLs are a group of heterogeneous lysosomal storage diseases characterized by the presence of an autofluorescent, pathologically derived material biochemically similar to the aging pigment lipofuscin (Seehafer and Pearce, 2006; Sedel et al., 2007). NCLs are classified into four different groups: infantile NCL, with symptoms onset between 6 months and 2 years of age and granular osmiophilic deposits in the skin, buffy coat, and other tissues (Wisniewski et al., 1992); late-infantile NCL, with onset between 2 and 4 years, and characterized by curvilinear deposits in similar areas as the infantile form (Wisniewski et al., 1992); juvenile NCL, the most common form, with onset between 4 and 10 years of age, and characterized by fingerprint deposits in neuronal and extraneuronal tissues (Wisniewski et al., 1992; Wu et al., 2014); and adult NCL (or Kufs disease), characterized by late onset (15 to 50 years) and characterized by mixed deposits primarily in neuronal tissue, with other tissues not always involved (Wisniewski et al., 1992, 2001). Studies over the past 20 years have identified 13 disease-related genes (Mole and Cotman, 2015) carrying loss-of-function mutations. Four types of NCL (CLN1, CLN2, CLN10, and CLN13) are caused by mutations in genes encoding for lysosomal enzymes, two (CLN5 and CLN11) by defects in soluble lysosomal protein, and five (CLN3, CLN4, CLN6, CLN7, and CLN8) by mutations in genes associated with lysosomal transmembrane protein (Kyttälä et al., 2006; Jalanko and Braulke, 2009; Mukherjee et al., 2019). CLN12 is caused by variants in *ATP13A2*, which encodes for a phospho-adenosine triphosphatase that actively transports inorganic cations, while mutations in a potassium channel gene are responsible for CLN14 (Kollmann et al., 2013).

**Figure 4:**
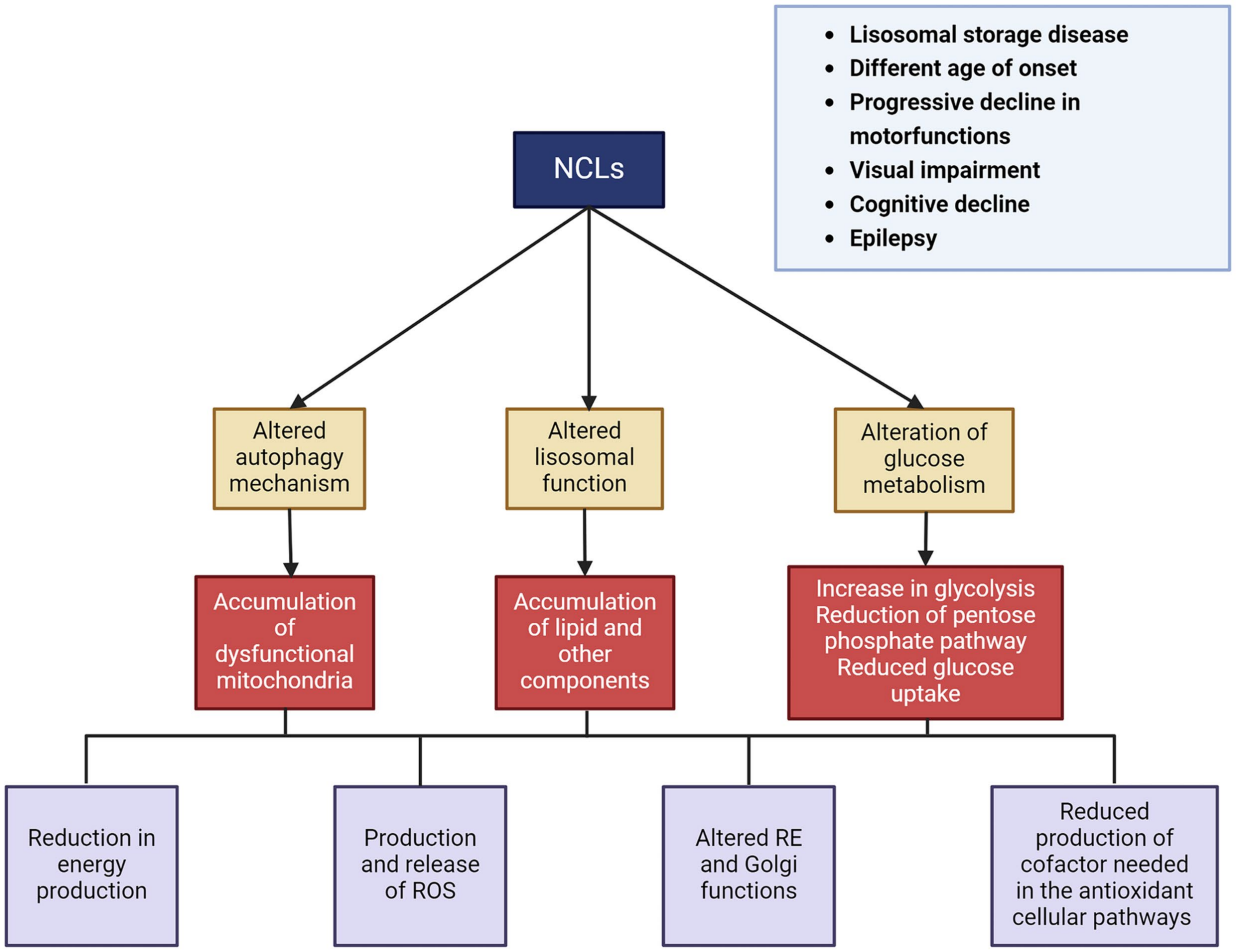
Principal clinical features and pathomechanisms of the NCLs.

Affected individuals initially show normal development in terms of cognitive function, movements, and vision. However, after an interval that varies depending on the type of disease, they begin to experience loss of vision and cognitive/motor impairments, followed by epilepsy (Wisniewski et al., 2001). At approximately 5–8 years of age, motor function declines, leading to an increasing loss of autonomy. Another characteristic feature of NCLs is progressive neuronal loss and eventually brain atrophy. Although the causes are not yet fully understood, this atrophy is probably the result of impaired lysosomal activity, which is also associated with the disruption of autophagy and other degradation pathways essential for cell survival. Although the disease eventually affects all of the gray matter, selective neurodegeneration can be observed in its early stages (Autti et al., 2008; Dyke et al., 2016). The cerebral and cerebellar cortices are the most affected brain regions, which is reflected in the major symptoms of the disease. Hippocampal degeneration has also been described in various forms of NCLs (Tyynelä et al., 2004). Additionally, this neuronal degeneration is associated with microglial and astrocyte activation, leading to the release of inflammatory cytokines and chemokines (Sanz and Serratosa, 2020) (Figure 4).

The pathological consequences of NCLs are most prominent in the central nervous system and the eyes. Neurodegeneration due to the accumulation of storage material is a mechanism shared by different forms of NCLs. This storage material resembles ceroid and lipofuscin, which also accumulates during normal aging (Marotta et al., 2017; Nita et al., 2016). The accumulation of storage material is linked to lysosomal activity impairment due to gene deficiency. Furthermore, dysfunction in neuronal lysosomal function leads to alterations in autophagy and other intracellular degradation pathways, which are essential for cell survival, inducing cell death (Darios and Stevanin, 2020; Kaminiów et al., 2022). The composition of the neuronal storage materials is still only partially defined, but the main components that accumulate include subunit c of the mitochondrial ATP synthase (SCMAS) and sphingolipid activating proteins saposins A and D (Simonati and Williams, 2022). During the last few years, several NCL proteins with previously unknown functions have been identified, most of which are lysosomal enzymes and proteins (Kollmann et al., 2013). It is important to note that lysosomes play a crucial role in sensing cytosolic glucose and glycogen availability. They also appear to be involved in glycogen autophagy (glycophagy) and lysosomal glucose transport, since lysosomes contain numerous glucose transporters (Mancini et al., 2023). There are still many unanswered questions about how lysosomes sense glucose and glycogen availability and how they regulate glucose uptake and release. A deeper understanding of lysosomal glucose metabolism is essential for uncovering the role of lysosomes in the pathogenesis of NCL disease.

### 5.1 Glucose metabolism in the NCLs

Evidence of hypometabolism in the NCLs was first observed in a disease very similar to human late-infantile NCL, which occurs naturally in English Setters. In this dog breed, symptoms such as brain atrophy, retinopathy, and cognitive decline begin to appear at approximately 13 months of age. [^18^F]DOG-PET scan showed reduced glucose uptake and diffuse hypometabolism in the brain (Kitani et al., 1995). Different studies in humans showed similar results. Fueki et al. (1990) examined a case of terminal-stage late-infantile NCL with severe seizures and constant myoclonus. PET brain scans revealed significantly reduced glucose metabolism in the cortex, along with cortical and subcortical atrophy, and diffuse hypometabolism. Philippart et al. (1997) conducted a PET analysis on four patients under 8 years of age with clinical symptoms of CLN2. This study also showed diffuse hypometabolism and brain atrophy. In addition, a lower concentration of insulin-like growth factor 1 (IGF1) has been found in the cerebrospinal fluid of patients affected by infantile NCL (Riikonen et al., 2000). IGF1 is important for early brain development, axonal growth, and myelination, and it also inhibits apoptosis (Riikonen et al., 2000). Interestingly, the motor neuron degeneration (mnd) mnd/mnd mouse, considered a model of CLN2 due to its pathological similarities with the human disease, showed partial recovery when treated with IGF1 (Cooper et al., 1999). De Volder et al. (1990) studied four siblings with juvenile or adult NCL, and [^18^F]DOG-PET scans again demonstrated reduced glucose utilization in the gray matter, particularly in the thalamus and posterior cortex. Similar results were obtained in another case of late-infantile NCL (Iannetti et al., 1994). Moreover, in Cln7^Δex2^ mice, a mouse model of CLN7 created by deleting exon 2 in the *Cln7/Mfsd8* gene (Brandenstein et al., 2016), levels of 6-phosphofructo-2-kinase/fructose-2,6-biphosphatase 3 were upregulated with a pro-glycolytic effect, suggesting a link to the human disease pathogenesis (Lopez-Fabuel et al., 2022). In a yeast model of juvenile NCL, created by deletion of btn1, an ortholog of CLN3, increased glycolysis, and altered amino acid metabolism were observed (Pears et al., 2010).

**Figure 5:**
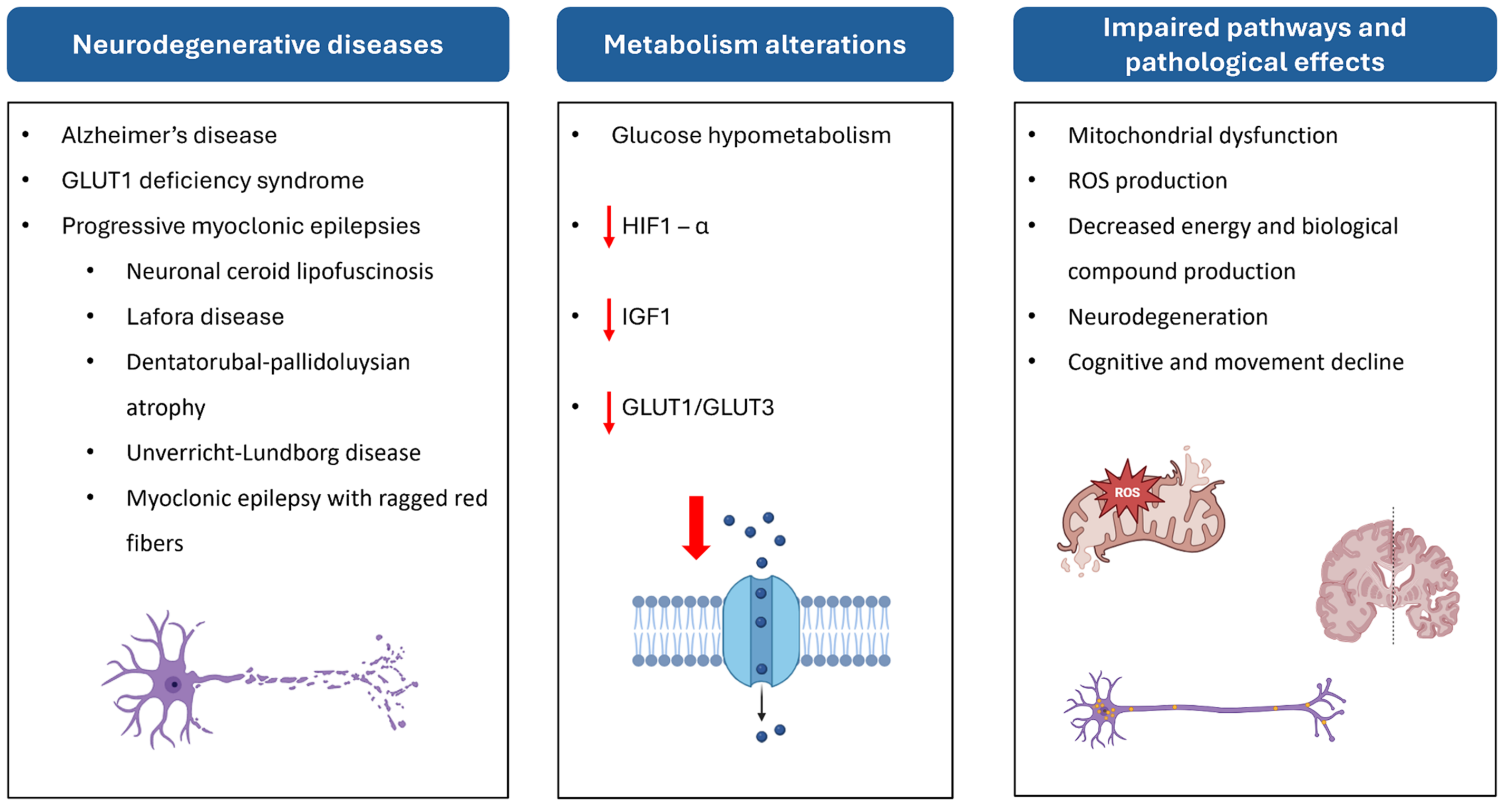
Scheme of the relationship between neurodegenerative diseases, altered metabolic pathways, and their effects at cellular and physiological levels. HIF-1α, hypoxia-inducible factor 1 subunit alpha; ROS, reactive oxygen species.

All this evidence, taken together, suggests that impaired glucose metabolism is closely associated with NCL pathogenesis and neurodegeneration (Figure 5).

## 6 Repositioning of anti-diabetic drugs as a new therapeutic approach to neurodegenerative diseases

The use of anti-diabetic drugs to treat neurodegenerative disease is a recent development. Different studies suggest that these drugs can improve cognitive impairment and reduce neuroinflammation, oxidative stress, and hypoglycemic activity in the brain (Birajdar et al., 2023). Anti-diabetic drugs have recently been used to treat various neurodegenerative diseases, such as AD, PD, and Huntington’s disease, with positive effects on disease progression (Sanz et al., 2021).

Metformin, a drug commonly used to treat type 2 diabetes mellitus, has demonstrated effectiveness against neurodegeneration *in vitro*, *ex vivo*, and in animal models by reducing oxidative stress, neuroinflammation, and neurodegeneration (Ristow, 2004). Since 2016, both the European Medicines Agency and the United States Food and Drug Administration have recognized metformin as an orphan drug for the treatment of LD due to its observed benefits, such as reducing neuronal loss and reactive astrogliosis in a mouse model and decreasing the length and number of seizures in treated patients (Berthier et al., 2016; Burgos et al., 2023). It is important to highlight that metformin activates AMPK and inhibits mammalian target of rapamycin (mTOR), via both AMPK-dependent and AMPK-independent pathways. Alterations of both AMPK and mTOR pathways are associated with neurological disorders (Foretz et al., 2014). Furthermore, metformin is also used to halt or delay the progression of epilepsy (Sanz et al., 2021). In this context, AMPK activation by metformin inhibited mTOR, leading to improved seizure control in a model of mTOR over-activation (Mehrabi et al., 2018). It is tempting to hypothesize that metformin might also serve to control symptoms in similar neurodegenerative conditions, such as NCLs. Furthermore, it is important to also consider glucagon-like peptide 1 (GLP-1) receptor agonists, which are a class of molecules used for the treatment of type 2 diabetes mellitus (Meier, 2012). They bind to a G-protein coupled receptor that activates adenylyl cyclase and phosphatidylinositol-3-kinase (Grieco et al., 2019). These enzymes produce secondary messengers that trigger various cellular processes, including cell survival, neuronal development, neurogenesis, autophagy, and mitochondrial function regulation (Grieco et al., 2019; Calsolaro and Edison, 2015; Smith et al., 2019). The use of GLP-1 receptor agonists in the treatment of neurodegenerative diseases is well-documented in the scientific literature, based on the neuroprotective effects that these drugs showed (Kopp et al., 2022; Reich and Hölscher, 2022). For this reason, there are some ongoing trials for neurodegenerative diseases such as Alzheimer’s or PD. The GLP-1 analog semaglutide (Ozempic) has shown neuroprotective effects in the 3xTg mouse model of Alzheimer’s disease. PET scans with [18F]DOG demonstrated a recovery of glucose metabolism in the brains of these mice (Wang et al., 2023). Based on these positive results, currently, two phase III clinical trial trials are testing Ozempic in AD patients (NCT04777396 and NCT04777409). Lixisenatide, another GLP-1 receptor agonist (Hölscher, 2024), has been used in a clinical trial for PD (NCT03439943). The results of phase II of this trial show great improvement in the Movement Disorder Society-Unified PD rating scale (MDS-UPDRS) test 2 months after the suspension of the drug treatment. These studies support our hypothesis that anti-diabetic drugs could play an important role in treating other neurodegenerative diseases, such as PME and NCLs. However, more studies are required. Improved characterization of impaired glucose metabolism in NCL patients could raise the prospect of a potential therapeutic approach able to delay or even halt disease progression in this population.

## 7 Discussion

Glucose is the primary energy source for neurons, supporting both signaling and non-signaling activities (Zhang et al., 2021). Glucose metabolism and brain functions are closely linked, as seen in conditions such as AD, LD, DRPLA, mitochondrial disease, and the NCLs, where glucose metabolism is reduced. Glucose metabolism also varies according to age, with the cerebral metabolic rate of glucose found to peak at 3–4 years (Chugani et al., 1987). This high metabolic rate may be one reason why NCLs, being childhood diseases, are associated with such devastating effects on the brain and cognitive development. Several studies have reported abnormal glucose metabolism in various NCLs, with most cases showing impaired glucose uptake by the brain. However, more research is needed to elucidate the cause of this abnormal glucose metabolism. Since a lower concentration of IGF1 has been identified in patients with infantile NCL, it might be interesting to examine its pathway defects. This growth factor promotes cell survival by inhibiting apoptosis and regulating neurogenesis and synaptogenesis at all stages of brain development, from embryonic through to adulthood (Nieto-Estévez et al., 2016). Targeting this pathway may offer better strategies for addressing key pathogenic mechanisms in NCLs, and other PMEs. The absence of specific data on impaired glucose metabolism in *in vitro* and *in vivo* models of the NCLs and other forms of PME may be due to a greater focus on molecular mechanisms such as autophagy, lipid metabolism, and neuroinflammation, which are already known to be associated with lysosomal dysfunction. It is important to note that the studies on glucose metabolism in NCLs identified in this review distinguish between the infantile, late infantile, juvenile, and adult forms, but fail to characterize the impaired glucose metabolism associated with each of these groups.

Moving forward, analyzing impaired brain glucose metabolism in experimental models (both animal and *in vitro* models) will be essential for discovering new molecular signatures of the NCLs and therefore novel drug targets. Data from studies on other neurodegenerative diseases (AD and PD) showed that compounds capable of restoring glucose metabolism to optimal levels can improve motor symptoms and prevent neurodegenerative decline (Nowell et al., 2023). For instance, in one clinical trial (NCT00620191), patients with AD treated with metformin showed a statistically significant improvement on the selective reminding test (Luchsinger et al., 2016). Another trial (NCT05781711), currently in the recruiting phase, will examine the efficacy of metformin in patients with PD, while trial NCT05532813 will test metformin in patients with myotonic dystrophy type 1, a condition associated with neurodegeneration and progressive muscular dystrophy (De Serres-Bérard et al., 2021).

**Figure 6:**
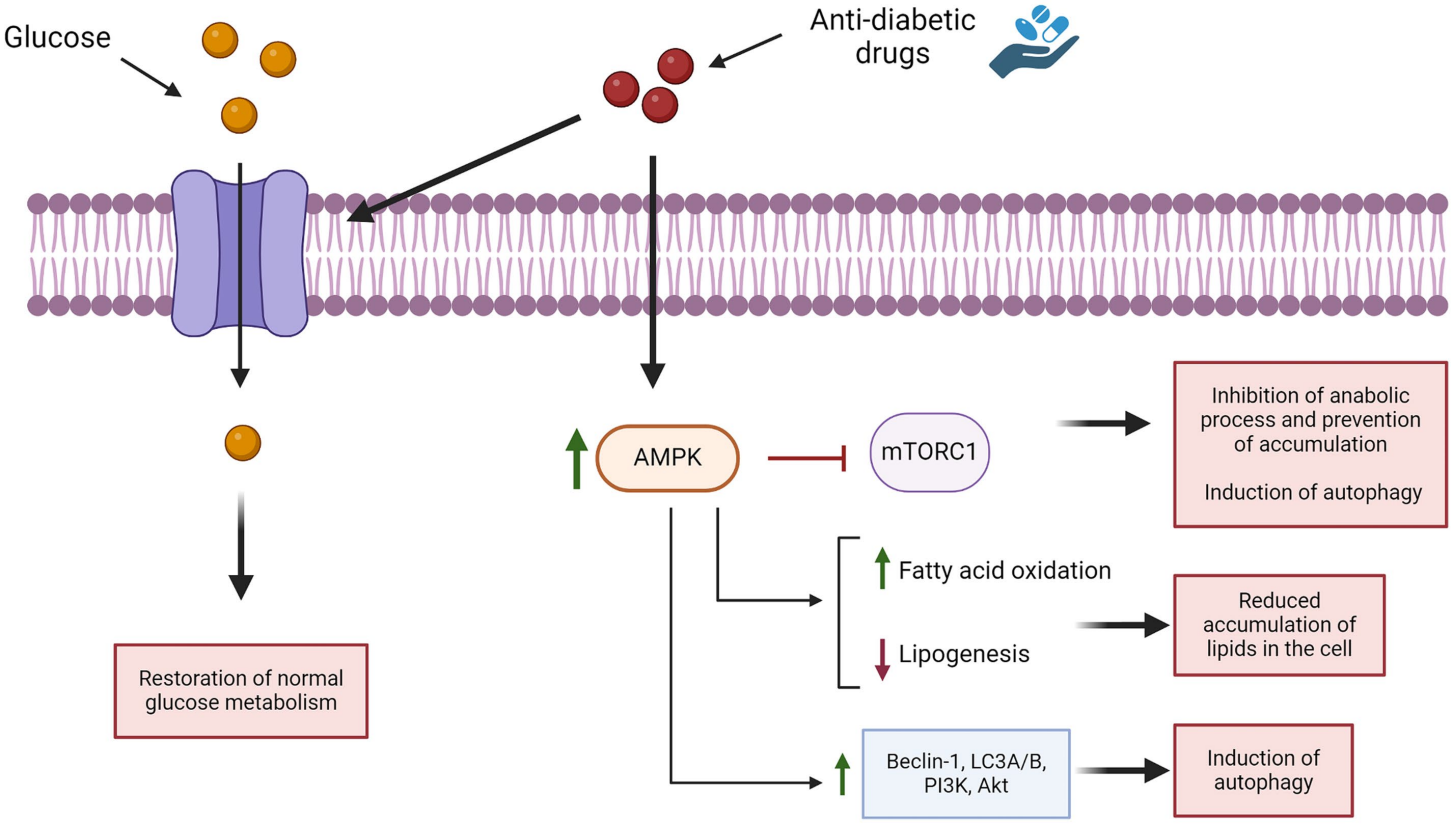
Effects of anti-diabetic drugs in pathways associated with NCLs.

NCLs are a heterogeneous group of neurodegenerative disorders characterized by disruption in lysosomal functions. This leads to the accumulation of various substrates within the cell and alters autophagy pathways, causing excessive cellular stress that leads to neuronal death and activation of inflammation (Sanz and Serratosa, 2020). Anti-diabetic drugs induce autophagy by stimulating AMPK, which inactivates mTOR and activates different autophagic pathways (such as Beclin-1, LC3A-B, and others), leading to increased autophagy (Ashrafizadeh et al., 2019) (Figure 6). These drugs also reduce intracellular lipids by enhancing fatty acid oxidation and decreasing lipogenesis (Ashrafizadeh et al., 2019; Zhou et al., 2015). *In vivo* model data suggest that anti-diabetic drugs such as metformin and liraglutide can improve cognitive function, as well (Ashrafizadeh et al., 2019). Various types of anti-diabetic drugs have the ability to ameliorate inflammation, either indirectly (normalizing hyperglycemia) or directly (suppressing the expression of pro-inflammatory cytokine) (Yaribeygi et al., 2019). These compounds also inhibit apoptosis and oxidative stress (Glotfelty et al., 2020). All these studies and analyses suggest that restoring brain glucose metabolism could be a promising therapeutic approach for NCLs. To date, there are no data on the treatment of NCLs with anti-diabetic drugs, except for one study on the use of IGF1 in a mnd/mnd mouse model, which partially restored interneuronal numbers and reduced hypertrophy in some subregions (Cooper et al., 1999). Therefore, it is worth evaluating the effects of these drugs in *in vitro* and *in vivo* models of the disease, which can lead to discovering new therapeutic approaches for this devastating disease. Additionally, *in vivo* data could help determine the appropriate dosage needed to achieve beneficial effects for future clinical use. However, a limitation to consider is that while anti-diabetic drugs may alleviate symptoms, they cannot fully cure the disease since they do not address the genetic root cause. Although anti-diabetic drugs are known to offer neuroprotective benefits, it remains uncertain at what stage of NCLs these effects might still counteract disease progression and provide therapeutic benefits. Therefore, it is crucial to identify new biomarkers of the disease that can be detected very early in the disease’s onset, allowing for timely intervention. It is also important to underline that anti-diabetic drugs could be used in association with other drugs already used for the treatment of NCLs; however, it will be necessary to test if these drugs can be administered together without negative effects.

In conclusion, better identification of the impaired glucose metabolism pathway in NCLs may open new avenues for evaluating the therapeutic potential of anti-diabetic agents in this population. Activating common pathological mechanisms, such as autophagy and energy regulation, through anti-diabetic drugs could prove to be a powerful tool in treating PMEs, including NCLs, by helping cells eliminate the pathological accumulation of different substrates.

## Data Availability

The original contributions presented in the study are included in the article/Supplementary material, further inquiries can be directed to the corresponding author.
