# Supplementary figures and images for: Glucose metabolism impairment as a hallmark of progressive myoclonus epilepsies: a focus on neuronal ceroid lipofuscinoses
Source: Front Cell Neurosci. 2024 Sep 19;18:1445003. doi: 10.3389/fncel.2024.1445003 (PMC11447523; doi:10.3389/fncel.2024.1445003)

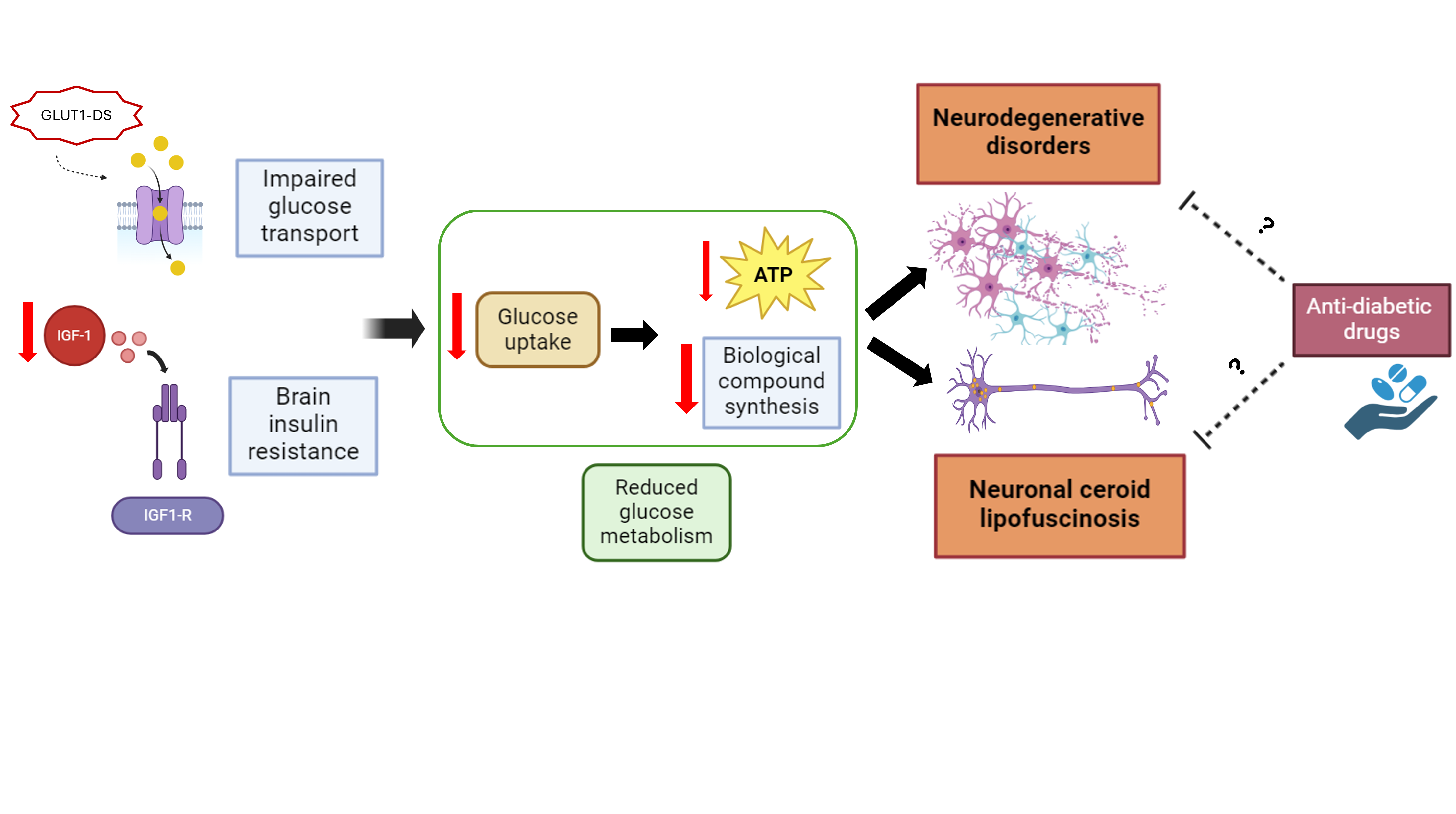

Supplement: Supplementary file 1 [file Image_1.TIF]
